# Six domains of health: a practical approach to identifying priorities in dementia care

**DOI:** 10.3389/frdem.2023.1188953

**Published:** 2023-06-22

**Authors:** Tatiana Sadak, Soo Borson

**Affiliations:** ^1^Biobehavioral Nursing and Health Informatics, School of Nursing, University of Washington, Seattle, WA, United States; ^2^Department of Family Medicine, School of Medicine, University of Southern California Keck, Los Angeles, CA, United States; ^3^Department of Psychiatry and Behavioral Sciences, School of Medicine, University of Washington, Seattle, WA, United States

**Keywords:** dementia, care complexity, multimorbidity, healthcare, care partner

## Abstract

**Background:**

High-quality healthcare for people living with dementia encompasses both patients and care partners (CPs). A framework populated with simple assessment tools is needed to deconstruct this complexity into actionable domains that inform assessment and care planning for individuals and dyads, help differentiate care team roles, and can more fully estimate true population burden in health and social care systems.

**Design:**

Researchers used a cross-sectional mixed-methods descriptive study to illustrate the use of an inductive Six Domain framework and simple assessment tools in a sample of dyads selected for complexity.

**Setting:**

Data was collected from three university-affiliated hospitals with a shared electronic medical record (EMR).

**Participants:**

Eighty-eight CPs for people living with dementia (care recipients) newly discharged home after an acute medical hospitalization participated.

**Measures:**

Care recipients' outpatient and inpatient diagnoses, medications, and care were extracted from the EMR. CPs completed an in-home semi-structured interview and study measures. Data were sorted into six domains: three care recipient-focused domains (cognition, emotion/behavior, general and functional health); a single CP-focused domain (mood, cognition, stress, and self-rated health); a health-related social needs domain (enrollment of persons with dementia in low-income insurance, CP-reported financial strain); and a care delivery domain (CP-reported engagement with clinicians in care recipients' care planning, and match between CP-reported knowledge of care recipients' medical care needs and medical records).

**Results:**

As expected, all people living with dementia had significant cognitive, neurobehavioral, and medical complexity requiring extensive oversight and management at home. Over a third of CPs reported high stress, depression, or anxiety. A fifth screened positive for one or more indicators of poor health, cognitive impairment, and/or health-related social needs. CP reports and care recipients' medical records were discordant for chronic conditions in 68% of cases and for prescribed medications in 44%. In 85% of cases, there were gaps in indicators of CP-clinician collaboration in care management.

**Conclusion and relevance:**

The Six Domains of Health framework captures a broad array of challenges that are relevant to providing comprehensive dyadic care and setting individualized health and social care priorities. With further study, it could provide conceptual scaffolding for comparative population research and more equitable, fully integrated pathways for care.

## Introduction

Alzheimer's disease and related dementias are complex, debilitating conditions that lead to progressive loss of agency, influencing health outcomes and how care is provided (World Alzheimer Report, 2022). Because most people living with dementia reside in the community (Yang et al., 2016), families and friends eventually become care partners (CPs), assuming healthcare responsibilities, organizing and sustaining relationships with clinicians, and delivering and monitoring prescribed, often complex, care plans at home (Reinhard et al., 2019). The crucial role of CPs in medical management is widely acknowledged, but its scope is not fully understood (Phelan et al., 2015; Reinhard et al., 2019), hindering the integration of CPs as full members of the healthcare team. Most tested interventions focus principally on helping CPs understand dementia, building skills to manage behavioral changes, and goal setting mainly within limited, typically dementia-specific domains (Gitlin et al., 2015; Phelan et al., 2015). Assisting CPs in practical understanding and managing patients' general medical care has received little attention (Phelan et al., 2015; The National Academy of Sciences Engineering and Medicine, 2019; Petrazzuoli et al., 2020) despite the nearly universal co-occurrence of several chronic conditions and a range of acute health risks in older adults who are living with dementia.

One-fifth of people living with dementia (care recipients) have six or more chronic conditions (Bunn et al., 2016), and hospitalization rates are two to four times higher than for comparable individuals without dementia (Phelan et al., 2012). As many as 90% of dementia CPs perform medical and nursing tasks, significantly more than CPs for older people living with other chronic conditions (National Alliance for Caregiving, 2017). Many feel alone and unsupported by clinicians (Reinhard et al., 2019). Many healthcare providers find caring for people with dementia difficult due to insufficient expertise, time, organizational resources, and staffing infrastructure to address the full range of needs (Mebane-Sims, 2020). In the United States, Medicare, the government-sponsored insurance plan that covers most older adults, provides a cognitive assessment and care planning benefit (Alzheimer's Association, 2019) introduced by the Centers for Medicare and Medicaid Services (CMS) in 2017. This benefit assumes good general medical care but does not specify what that entails in the context of dementia, nor does it support health system redesign or the changes in infrastructure necessary to provide it.

Furthermore, randomized dementia care management trials find no significant effect on a widely used indicator of care quality - hospitalization rates (Phelan et al., 2015; Frost et al., 2020). They offer little insight into how CPs deal with medical management at home or what coaching they need from clinicians (Reinhard et al., 2019). A simplified organizing framework that parses the clinical complexity of dementia dyads into distinct domains could help people living with dementia, CPs, and clinicians identify and track needs to be prioritized for management over time. Such a framework could eventually be embedded in healthcare information systems.

Informed by Watson's Theory of Caring Science: Social Justice and Human Caring (Watson, 2018), we expanded a four-part dementia care framework developed inductively by Borson (Lessig et al., 2006; Borson and Chodosh, 2014) from experience in routine, family-focused, longitudinal healthcare management of people living with dementia. The approach extends existing management models embedded in ambulatory healthcare settings (Hovsepian et al., 2022). To accommodate advances in research, two new domains (health-related social needs and care delivery factors) have been added to the initial four (care recipients' cognitive, emotional/behavioral, and physical health and function, and CP capacity and needs); each domain is associated with significant clinical, caregiving, and service utilization outcomes. The resulting Six Domains of Health framework reflects dimensions important in healthcare for people living with dementia and recognizes the dependence of successful healthcare on the capabilities and knowledge that CPs contribute. Consistent with the Theory of Caring Science, which seeks to integrate context and environment into healthcare delivery to provide truly person-centered care, the person living with dementia/CP dyad is seen as the unit around which care must be planned, delivered, and evaluated. To become testable, however, the framework must be practicable. For this preliminary study, we selected simple measures to populate each domain. The result is a profile of existing clinical complexity in a group of dyads identified soon after the home discharge of people living with dementia from an acute, unplanned medical hospitalization, a point at which care needs are likely to be particularly complex.

## Materials and methods

### Setting and participants

In three university-affiliated hospitals with a shared electronic medical record (EMR) system, we used an EMR algorithm to identify, within seven days of discharge, individuals with a diagnosis of dementia and at least two other chronic conditions who had been admitted from home to an acute general medical ward and had an identifiable next of kin (*n* = 446, identified over a 9 month period). We enrolled 88 CPs after excluding those who declined, did not respond after up to three telephone contact attempts, or were not the primary CP (i.e., did not provide regular hands-on care at home, participate in care recipients' medical appointments, or have authority to permit access to their medical records). All enrolled CPs had healthcare power of attorney and confirmed that their care recipient exhibited signs of dementia on the proxy-reported AD-8 (Galvin et al., 2005).

### Study procedures

CP provided written informed consent to participate in the study and written authorized access to care recipients' medical records. Although many people living with dementia, especially in the early stages, can report accurately on themselves, we selected CPs as the active study participants, and extracted clinical information about people living with dementia from their EMR to maintain methodological consistency across varying levels of cognitive impairment. CPs completed study measures and a 90-minute in-person interview that was recorded and transcribed by the researchers under a protocol approved by the University of Washington Institutional Review Board (Study #00002012). Interviewers were experienced Masters level clinicians; the primary investigator (TS) monitored fidelity by reviewing at least 25% of all recordings and providing feedback to the interviewers. The senior investigator (SB) reviewed a 10% sample of records and assisted in interpreting ambiguous information. Participants received $50 gift cards after completing the study.

## Data acquisition

### CP interviews

Participants were asked to prepare by collecting all available information regarding the most recent hospitalization, encounters with the care recipient's healthcare providers, and care recipients' prescribed medications and medical problems. They were encouraged to use notes, medical records, medication lists, and/or containers to assist recall. They were asked if any healthcare provider had ever told them about the dementia diagnosis, discussed what to expect, prepared them for how their responsibilities could change as dementia progresses, or referred them to geriatric or palliative care. They were encouraged to describe engagement in any primary care-based planning process, as well as any conversations about the pros and cons of hospitalization. CPs responsible for medication management (82/88) were asked to use all available aids to list care recipients' current prescribed medications (excluding supplements and topicals) and describe their purpose.

### EMR data

We summarized inpatient and outpatient records, including phone contacts, for the 30 days before index hospitalization, the inpatient period, and the interval from discharge to interview. Pre-hospitalization outpatient encounter notes were searched for documentation of any CP need (e.g., “stressed, needs respite,” “CP asked about…”) and any dementia-related care plan. Hospital discharge notes were reviewed for dementia-related recommendations. To capture the most accurate information about persons-living-with-dementia's chronic conditions, including dementia type if specified, we reviewed information from the most recent inpatient and outpatient notes, lists of active problems, discharge diagnoses, and CMS billing codes for the index hospitalization. Before the interview, we reconciled lists of current medications and medication orders from the most recent clinical encounter to identify prescribed medications.

### Measures

To populate clinical profiles across the health domains for each person living with dementia/CP dyad, persons-living-with-dementia's demographics and medical information were collected from the EMR. CPs completed a range of measures not available in the EMR. These are listed below.

#### Cognitive health and function

The severity of care recipients' cognitive impairment was assessed with the Dementia Severity Rating Scale (DSRS, score range 0-54, Cronbach's α = 0.92), a validated 12-item caregiver-rated dementia staging checklist (Moelter et al., 2015). Impairment in higher-order, cognition-dependent activities of daily living (IADL) was assessed with an 8-item checklist (UCLA Alzheimer's and Dementia Care Program, 2023).

#### Behavioral emotional health and function

The number of care recipients' challenging behaviors was scored using BEHAV5+ (score range 0–6), which captures behaviors most distressing for the CP; scores of 2+ are associated with unmet healthcare and social service needs (Borson et al., 2014).

#### Physical health and function

Care recipients' comorbidity, assessed via EMR was rated with the Charlson Comorbidity Index (CCI score range 0-33, including 17 acute and chronic conditions associated with hospitalization and mortality risk) (Charlson et al., 1987). The CMS Chronic Condition Warehouse (CCW) checklist of 27 common chronic diseases (Medicare Chronic Conditions Data Warehouse, 2023). Prescribed medications were identified via fully reconciled EMR records (hospital admission and most recent outpatient visit prior to the CP interview). Potentially inappropriate medications were identified by the Nurse Practitioner study staff using the Screening Tool for Older Person's Prescriptions (STOPP) (Gallagher et al., 2008). Everyday physical care dependency (yes/no) was assessed via the CP report with a 7-item basic Activities of Daily Living (ADL) checklist (UCLA Alzheimer's and Dementia Care Program, 2023). The CP reported the prior year's care recipients' acute care utilization (ED and inpatient).

#### Care partner capacity and needs

CPs' current mental health was assessed using the Patient Health Questionnaire-2 (PHQ-2, score range 0-6, Cronbach's α = 0.77) (Staples et al., 2019) for depression and the Generalized Anxiety Disorder-2 Scale (GAD-2; score range 0-6, Cronbach's α = 0.92) (Staples et al., 2019) for anxiety. Possible cognitive impairment was assessed by Mini-Cog (score range 0-5, Cronbach's α = 0.82) using the cut-point ≤ 3/5 (Borson et al., 2003). A single-item stress question (score range 1–5) was used to determine global CP stress; scores of 3+ are associated with unmet health and social care needs (Borson et al., 2014). The CP's overall health was indexed by the self-reported chronic conditions [CCW (Medicare Chronic Conditions Data Warehouse, 2023)] and a single-item global self-report (scored 1–5) (Bowling, 2005).

#### Health related social needs

Two indicators were used for initial screening: care recipients' enrollment in Medicaid, the US low-income insurance plan, and/or CP-reported difficulty paying for care recipients' basic needs (single-item rating scored 1-5, with 2+ representing at least some) (Gitlin and Rose, 2014).

#### Care delivery framework

We included two indicators of CP-clinician partnership and communication: CP perception of engagement in care planning and match between CP reported knowledge and EMR documentation of care recipients' medical conditions and treatments. We also included a general assessment of services available to people living with dementia and CPs within the health system (e.g., chronic care management, care coordination, assistance with transitions between care settings [e.g., hospital to home], referrals to specialists [e.g., geriatric, palliative, dementia], and CP support).

### Analyses

#### Descriptive analyses

Simple summary statistics characterized people living with dementia and CP across the six domains.

#### Calculating match between CP reports and EMR records of care recipients' current chronic conditions and medications

We explored concordance between CP reports and information available to the patient's provider based on EMR data (Schneider et al., 2009). To allow for a margin of error, we chose a threshold of ≥ 80% match to classify cases as concordant [International Society of Pharmacoeconomics and Outcomes Research ISPOR Medication Compliance Special Interest Group (Med Comp), 2012]. Medication lists prepared by the CP were matched against the current, fully reconciled EMR as the reference standard unless the CP explicitly reported provider-initiated discontinuation. We calculated concordance for all prescribed medications in aggregate and separately for cognitive enhancers and other psychotropic medications. For medicines identified by the CP, knowledge of medication purpose was evaluated by research nurse practitioners who reviewed interview transcripts and medical records, assigned a match if the purpose was identified correctly, and flagged potentially inappropriate medications (Gallagher et al., 2008). We used similar methods to assess concordance between CP and EMR for chronic conditions. We reviewed admission and discharge summaries and Medicare discharge diagnosis (billing) codes to evaluate whether discordance for medications and/or chronic conditions might have contributed to index hospitalization.

## Results

### Demographics

CPs were mainly adult children and spouses who had cared for a person living with dementia for an average of 5 years; the majority were women and college-educated, and over half lived with their care recipient. Care recipients, with a mean age of 83, included similar numbers of both genders (Table 1).

**Table 1:** Demographics.

	**Number (%) or Mean (sd), Range**
**Care partners (CP)**	
Age, mean (sd), range	64 (12.5), 33-88
Gender, female, *n* (%)	57 (65%)
Hispanic or Latino, *n* (%)	2 (2%)
**Race, or origin** ***n*** **(%)**	
Hispanic or Latino, *n* (%)	2 (2%)
White	73 (83%)
Asian	11 (13%)
African American/Black	3 (3%)
American Indian/Alaska Native	1 (1%)
**Education**, ***n*** **(%)**	
Graduate or professional degree	21 (24%)
College degree or vocational training	54 (61%)
High school	13 (15%)
**Relationship to person with dementia** ***n*** **(%)**	
Spouse/domestic partner	44 (50%)
Adult child	35 (40%)
Friend/other relative	9 (10%)
Live with a person with dementia, Yes, *n* (%)	47 (53%)
Years as care partner, mean (sd), range	5 (4.1), 0-16
**People living with dementia**	
Age, Mean (sd), range	83 (8.4), 62-98
Gender, female, *n* (%)	42 (48%)
Hispanic or Latino, *n* (%)	2 (2%)
**Race, or origin** ***n*** **(%)**	
Hispanic or Latino, *n* (%)	2 (2%)
White	72 (82%)
Asian	11 (13%)
African American/Black	4 (4%)
American Indian/Alaska Native	1 (1%)

*N* = 88 dyads.

### Profiles of clinical complexity. The six domains of health

#### Cognitive health and function

Two-thirds of care recipients had moderate to severe dementia; about half had a non-specific dementia diagnosis, with Alzheimer's disease being the most frequent among classified causes. Fewer than a quarter had a currently prescribed cognitive-enhancing medication; among those, a third of CPs failed to report its use or purpose. On average, people living with dementia had seven out of eight impaired IADLs (Table 2).

**Table 2:** Clinical complexity of people living with dementia and their care partners.

**Cognitive health and function**	**Number (%) or Mean (sd)**
**People living with dementia (care recipients)**	
Stage/severity of cognitive impairment	
Mild dementia (DSRS range 4–18)	29 (33%)
Moderate-severe dementia (DSRS range 19–48)	59 (67%)
Number of impaired IADL [0–8, mean (sd)]	7 (1.5)
**Cognitive impairment diagnosis (ICD-10; 1 or more)**	
Dementia not otherwise specified	42 (48%)
Alzheimer's disease	26 (30%)
Vascular Dementia	16 (18%)
Parkinson's disease with dementia or dementia with Lewy Bodies	13 (15%)
Frontotemporal	2 (2%)
Prescribed a cognitive enhancer (dementia) medication (1 or more)	21 (24%)
No dementia-related care notes in outpatient EMR (*N* = 71 care recipients who had an outpatient care visit within 30 days prior to index admission)	56 (79%)
**Care partners (CP)**	
CP did not report or identify the purpose of prescribed cognitive-enhancer medication (*N* = 21 care recipients with relevant prescriptions)	6 (29%)
**Behavioral emotional health and function**	**Number (%) or Mean (sd)**
**People living with dementia (care recipients)**	
Depression diagnosis in EMR	35 (40%)
Number of difficult behaviors (BEHAV5+, mean, range 0–6)	3 (1.7)
Indifference/social withdrawal	48 (55%)
Agitation/aggression	46 (52%)
Irritability/frequently changing mood	45 (51%)
Sleep problems	35 (40%)
Hallucinations	24 (27%)
Suspiciousness/paranoia	22 (25%)
2+ difficult behaviors	57 (65%)
Currently prescribed 1+ psychotropic medication (excluding cognitive enhancers)	36 (41%)
Delirium or altered mental status on index admission	31 (35%)
**Care partners (CP)**	
Did not report prescription or purpose of psychotropic medication excluding cognitive enhancer (*N* = 36 care recipients with relevant prescription)	14 (38%)
**Physical health and function**	**Number (%) or Mean (sd)**
**People living with dementia (care recipients)**	
Charlson Comorbidity Index (EMR), (0-33)	9 (2.9)
Number of chronic conditions (CCW, EMR), (0-27)	8 (2.7)
Number of impaired ADLs (0-7)	3 (2.6)
Number of medications (EMR)	7 (3.7, 0–16)
At least 1 STOPP medication prescribed (EMR), persons (%)	52 (59%)
Hospitalizations & Emergency Room visits, past 12 months (CP report)	4 (3.1, 0–15)
**Care partner needs and capacity**	**Number (%) or Mean (sd)**
**Care partner (CP) health**	
Depression (PHQ-2 score 2+/6)	27 (31%)
Anxiety (GAD-2 score 3+/6)	28 (32%)
Possible cognitive impairment (Mini-Cog 0-3/5)	18 (21%)
Moderate to severe stress (Stress thermometer score 3+/5)	34 (39%)
Number of chronic conditions (CCW, CP report)	2 (2.1)
**Perceived health**	
Very good or good	70 (80%)
Fair or poor	17 (19%)
**Health related social needs**	**Number (%) or Mean (sd)**
Financial strain—CP reports difficulty paying for basic needs of person living with dementia	36 (41%)
Person living with dementia is Medicaid recipient	15 (17%)
**Care delivery framework**	**Number (%) or Mean (sd)**
CP did not recall ever discussing care recipient's dementia diagnosis with healthcare provider	26 (29%)
CP reported no history of referral for care recipient to geriatric or palliative care services	48 (59%)
No dementia care recommendations in hospital discharge notes	73 (83%)
No dementia-related care notes in outpatient EMR (*N* = 71 people living with dementia who had an outpatient care visit within 30 days prior to index admission)	56 (79%)
No mention of CP's needs in outpatient EMR (*N* = 71 people living with dementia who had an outpatient care visit within 30 days prior to index admission)	60 (85%)
**Discordance between CP report and EMR**	
< 80% match of care recipient's medications (*N* = 84 people living with dementia had active prescriptions)	37 (44%)
< 80% match of care recipient's active chronic conditions	60 (68%)
Gaps in CP knowledge of medication purpose, missed 1+ (*N* = 84 people living with dementia had active prescriptions)	25 (30%)
Chronic condition missed by CP was a documented cause or contributing factor for the index hospitalization of the care recipient	14 (16%)
Medication missed by CP was indicated for a diagnosis that contributed to the index hospitalization of the care recipient	9 (10%)

*N* = 88 dyads, unless otherwise stated.

EMR, Electronic Medical Record; CCW, Medicare Chronic Condition Warehouse inventory of chronic conditions; DSRS, Dementia Severity Rating Scale; BEHAV5+, list of 6 behavioral problems; Stress thermometer, single-item analog scale for CP stress; STOPP, inventory of medications not recommended for older adults; PHQ-2, 2-item Patient Health Questionnaire (depression); GAD-2, 2-item Generalized Anxiety Disorders questionnaire; Mini-Cog, cognitive impairment screener; ADL, basic activities of daily living; IADL, instrumental activities of daily living.

#### Behavioral emotional health and function

Over a third of care recipients were diagnosed with altered mental status or delirium on index hospitalization. After discharge home, they had an average of three challenging behaviors (most common were indifference/social withdrawal, agitation/aggression, and irritability), and 40% had a diagnosis of depression in the EMR. Among care recipients with any psychotropic medication prescription other than a cognitive enhancer (41%), over a third of CPs failed to report it or could not state its purpose (Table 2).

#### Physical health and function

People living with dementia had a mean of three out of seven impaired ADLs, a Charlson Comorbidity Index of nine, eight chronic conditions, seven post-discharge medications, and four previous acute care episodes in the year before their index hospitalization. Over half of people living with dementia were prescribed at least one STOPP medication (Table 2).

#### Care partner capacity and needs

Nearly 40% of CPs reported moderate to severe stress, about a third screened positive for depression and/or anxiety, a fifth rated their overall health as fair or poor, and a fifth screened positive for cognitive impairment (Table 2).

#### Health related social needs

Seventeen percent of people living with dementia were dually enrolled in Medicare and Medicaid, and 41% of CPs reported difficulty paying for basics for persons-living-with-dementia's care (Table 2).

#### Care delivery framework

All recruitment sites were part of UW Medicine, the northwest US's only comprehensive clinical, research, and academic health system in the five-state WWAMI region encompassing Washington, Wyoming, Alaska, Montana, and Idaho. Although this health system includes an extensive network of primary care clinics and several hospitals, we found that opportunities for care coordination/transitions and CP support are few: referrals to specialty geriatric, palliative, and dementia services are limited, and no formal dementia care pathway has been implemented across the system.

The discharge planning process for care recipient/CP revealed gaps: no dementia-related care recommendations or evidence of CP coaching were found in 83% of hospital discharge notes. We also identified gaps in the outpatient care provided within 30 days before the index hospitalization. For the 71 people living with dementia with at least one outpatient visit, most had no dementia-related care plan referenced in records, and 85% of documented encounter notes did not mention CP's needs.

There were also gaps in indicators of CP-clinician partnership: a third of CPs reported never being told by a healthcare provider about the care recipient's dementia diagnosis. Most CPs did not recall conversations about how to prepare and what they need to know to manage care recipients' health as dementia progresses, and 40% did not recall ever being asked to participate in identifying care priorities. Among 75 CPs who had contact with the care recipient's outpatient provider before index hospitalization, about 60% could not recall any discussion of the pros and cons of hospitalization (Table 2).

**Figure 1:**
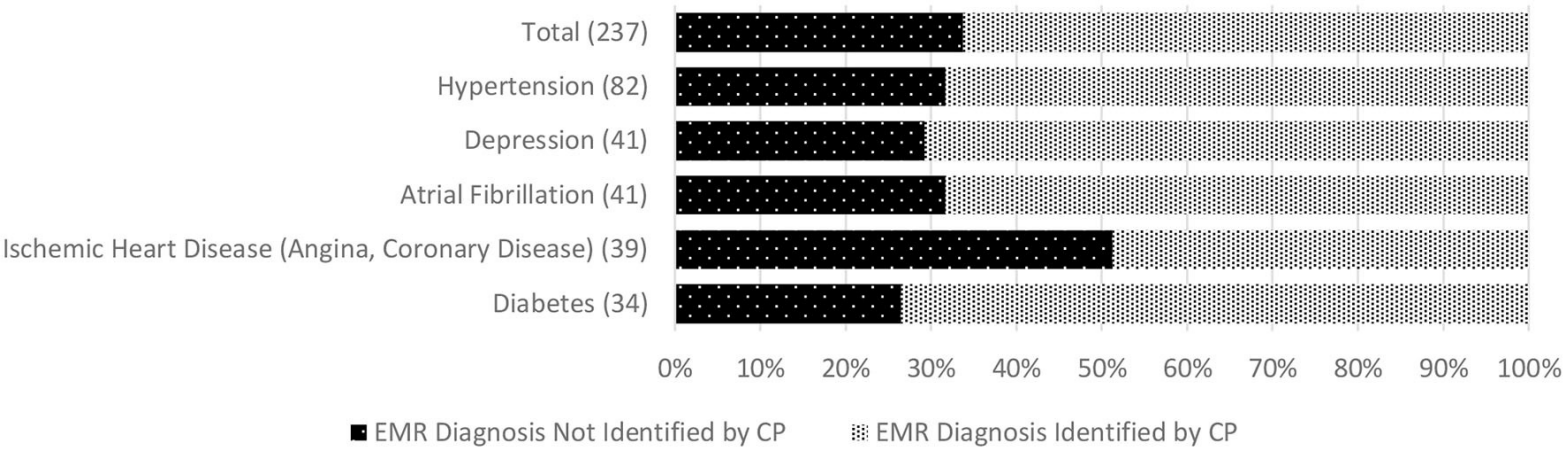
Discordance between CP report and Electronic Medical Record of person living with dementia (care recipient's) chronic condition diagnoses (based on 80% match). *Numbers in parentheses indicate the total number of people living with dementia with each selected chronic condition across the whole sample (top graph). EMR, Electronic Medical Record; CP, Care Partner.

**Figure 2:**
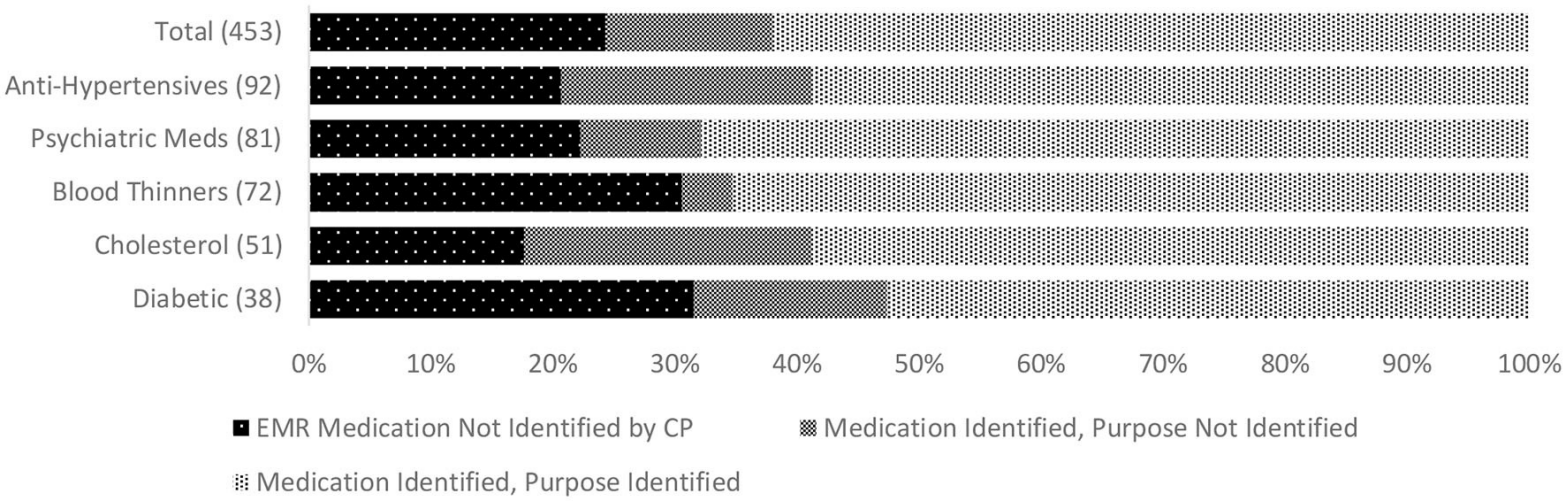
Discordance between CP report and EMR record of people living with dementia who were prescribed medications (based on 80% match) and identification of the purpose of prescribed medications by CP. *Numbers in parentheses indicate the total number of prescribed selected types of medication across the whole sample (top graph) for people living with dementia. EMR, Electronic Medical Record; CP, Care Partner.

We found additional evidence of inadequate CP-clinician communication related to care recipients' ongoing medical management. This included low concordance for active chronic conditions among 68% of CPs. In 16%, a condition not recognized by the CP was a documented cause or contributing factor for the index hospitalization. The most commonly unreported chronic conditions were hypertension, depression, atrial fibrillation, ischemic heart disease, and diabetes. Low concordance for medications was found among 44% of CPs, and for medications correctly identified, 30% of CPs could not state the purpose of at least one. In 10%, an unreported medication was indicated for a diagnosis contributing to the index hospitalization. Among the medicines most commonly missed, or misidentified by indication, were prescriptions for diabetes, hypertension, hypercholesterolemia, anticoagulation, and psychotropics. Figure 1 depicts concordance between the EMR and CP reports of chronic conditions and lists conditions most often missed by CPs. Figure 2 illustrates the concordance between the EMR and CP reports for medications and lists those most often overlooked by CPs.

## Discussion

We demonstrate how a simple Six Domains of Health framework, populated with brief assessments and easily automated EMR data extraction, can help assess and organize the care management needs of dementia care recipient/CP dyads. The resulting snapshot lets us identify the most urgent priorities without losing sight of overall dyadic complexity. We also illustrate how the Six Domains of Health framework offers the information infrastructure needed to operationalize the Theory of Caring Science in dyadic dementia management. Comprehensive information is necessary but not sufficient. Care planning clearly requires identifying and reconciling priorities for all key partners, including members of the interdisciplinary clinical team, people living with dementia, CPs, and their wider support network. Two hypothetical examples illustrate action plans based on information from a systematic assessment of the Six Domains of Health (abbreviated below as COG [cognition], BEH [behavior], PHY [physical health], CP [CP capacity/needs], HRSN [health related social needs], CDF [care delivery framework]).

### Case 1

Mr. D, age 83, has moderate-stage dementia (COG) and was hospitalized for exacerbations of heart failure (PHY) and agitation (BEH) twice in the last 6 months. His primary CP is his cheerful, talkative 84-year-old wife. Mrs. D doesn't realize his ankle swelling is caused by heart failure and can't explain why he takes furosemide (CP)—she, too, has some cognitive impairment (CP) but denies stress, depression, and anxiety. With Mrs. D's permission, a healthcare provider calls their daughter, who lives nearby—she checks Mr. D's medication organizer and finds he has missed half of his diuretic doses (PHY). When the clinician explains both parents' needs, the daughter agrees to help oversee medications and attend future appointments. Still, due to her busy schedule, she also plans to engage a home health nurse consultant (CDF). The daughter also agrees to enroll in a dementia support and education group with her mother (CDF).

### Case 2

Mrs. P, age 87, lives alone with mild dementia (COG), moderate COPD, and chronic kidney disease (PHY), but her sister, age 80, lives close by, visits often, and assists as needed. Sister is unaware of Mrs. P's dementia diagnosis and does not accompany her to medical appointments (CP). Mrs. P. has Medicare hospitalization and outpatient visit benefits but no insurance coverage for medications (HRSN). A month ago, she had multiple ER visits (CDF) and was hospitalized for pneumonia, which led to acute kidney injury (PHY). She understands her medical problems fairly well but confides that fatigue makes it hard to manage everything. The healthcare provider notices that two of her daily COPD medications are expensive. Mrs. P. admits she saves them 'for emergencies' because of cost (HRSN, CDF). She denies significant stress, anxiety, and depression but reports feeling lonely for her kids (BEH), who live out of state. After a brief consult with a pulmonologist and nephrologist to identify additional treatment needs (PHY), the patient receives more affordable medications (PHY, CDF). The clinic social worker (CDF) holds a virtual family conference (PHY, BEH, CP) that results in a move to the patient's daughter's town.

As a formative investigation, our study is limited by its conceptual, descriptive, non-interventional design and modest assessment of the newly added domains of the health-related social needs and care delivery framework. Our relatively small, high-risk sample does not represent diverse populations or most care recipient/CP dyads who live in the community, due to patients' fairly advanced dementia and complex morbidity. Selection bias in recruitment is likely, so the distribution of problems identified by study measures cannot be taken as representative or generalizable. We purposely recruited for complexity, while the majority of people living with dementia in the community would be expected to have less complex overall profiles. Finally, we did not capture the lived experiences of people living with dementia; in a population sample, many individuals will be in earlier stages of dementia and could be engaged in a conversation about their own needs and preferences.

Future studies should evaluate the full scope of health-related social needs, including housing instability, food insecurity, transportation problems, utility help needs, interpersonal safety, physical safety, family/community support, physical activity, and substance use, as influences on care delivery. Assessment of care delivery factors was similarly minimal in this study; additional relevant care delivery factors need to be considered based on the evolving science and the setting in which care is being provided. They may include the attributes of age-friendly health systems (e.g., care navigation, coordination, transitional, telemedicine, and home-based primary care services; advanced care planning and serious illness conversation-related documents in the EMR; transparency about services covered by the patient's insurance plan used to optimize affordability), special emphases on access and equity (e.g., accommodation for language, hearing, vision, mobility or other difficulties; information, support, & resources for financial, legal, and long-term care planning tailored to language, education, and culture; information about modifiable risk factors for poor outcomes (e.g., uncompensated hearing/vision loss, social isolation, physical inactivity, persistent depression, poorly managed comorbid conditions). Future studies should evaluate the proposed multi-domain framework as a guide to re-engineering dementia care in all settings, with innovations that enhance the value of care, optimize health outcomes, refocus on the person/support system, and make dementia care easier to do well (Bott et al., 2019).

Randomized trials of dementia care management, provided by experts within healthcare settings (Vickrey et al., 2006; Callahan et al., 2012; Reuben et al., 2019) or in the community (Schulz et al., 2003; Tanner et al., 2015), have demonstrated improvements in indicators of dementia care quality and CP stress and wellbeing. Similar advances have not occurred in primary care, where most dementia is diagnosed and most patients receive care. In its 2019 systematic review of care interventions for individuals with dementia and their CPs, the National Academies of Science, Engineering, and Medicine identified a paucity of evidence-based interventions that address medical complexity in the context of dementia and engage and support CPs in providing medical management of people living with dementia at home (The National Academy of Sciences Engineering and Medicine, 2019). The report also noted a dearth of ways to identify CP knowledge gaps, concluding that the optimal dementia care of the future will be based on tailored interventions that combine tested strategies based on the analysis of individual gaps and needs. Our work confirms these gaps and takes a step toward filling them: the Six Domains of Health framework is a straightforward approach for assessing dementia dyads' needs and building practical person-CP-centered care plans and intervention strategies. We focus on unifying into a single framework the broad range of challenges that patients, families, clinicians, and health systems face as they work toward becoming “dementia capable” (Borson and Chodosh, 2014). Our previous reports on using the partial framework to identify and drive individualized interventions in the actual clinical care (Lessig et al., 2006), and our descriptive study in an advanced illness care innovation embedded in a health system, further validate the relevance of the framework for CPs of older patients with and without dementia (Borson et al., 2018). Achieving real improvement in health outcomes in persons living with dementia must integrate such competencies into all care (Heintz et al., 2020; Tuzzio et al., 2020).

Although our study used data from a vulnerable subset of individuals who were living with dementia and multimorbidity and experienced a major acute illness requiring hospitalization, the Six Domains of Health framework flexibly accommodates a broad spectrum of clinical heterogeneity and sets the stage for standardizing expectations for clear, practical conversations around goals, CP coaching needs, and critical action steps to manage persons living with dementia health effectively at home. This framework could guide the care of frail older people or those living with multi-morbidities regardless of dementia diagnosis and care setting. Another important finding, supported by the existing literature, is that many care partners experience significant stress and physical, cognitive, and emotional challenges that add a layer of complexity and require assessment and care planning (The National Academy of Sciences Engineering and Medicine, 2019; World Alzheimer Report, 2022).

For maximum impact, the Six Domains of Health framework must find a home within a care delivery framework that strives to become person-family centered and dementia capable. When used to guide practice improvement efforts, it can help create the human and health information infrastructure that makes comprehensive dementia care feasible, actionable, and measurable. When used as a guiding principle by the responsive, dementia-capable health systems, the Six Domains of Health framework can help implement whole-person, trustworthy, transparent, proactive, equitable, flexible, relevant, specific, goal-directed care that is optimized for improving quality of life, and responsive to psychosocial and spiritual needs of all stakeholders.

## Data availability statement

The raw data supporting the conclusions of this article will be made available by the authors, without undue reservation.

## Ethics statement

The studies involving human participants were reviewed and approved by the University of Washington IRB. The participants provided their written informed consent to participate in this study.

## Author contributions

SB created the original 4-part and newer 6-part model framework. SB and TS conceptualized the study reported here and collaborated in analyzing and interpreting data and writing the manuscript. TS served as the study PI. All authors contributed to the article and approved the submitted version.
